# Associations Among Knee Osteoarthritis Severity, Body Mass Index, and Physical Functions in Saudi Arabian Adults: A Multi-Center Cross-Sectional Study

**DOI:** 10.7759/cureus.48130

**Published:** 2023-11-01

**Authors:** Vishal Vennu, Ali D Al-Otaibi, Saud A Alfadhel, Saad M Bindawas

**Affiliations:** 1 College of Applied Medical Sciences, King Saud University, Riyadh, SAU; 2 Physical Therapy, Dawadmi General Hospital, Dawadmi, SAU; 3 Physical Therapy, General Directorate of Medical Services, Riyadh, SAU; 4 Rehabilitation Sciences, King Saud University, Riyadh, SAU

**Keywords:** physical function performance, body mass index, knee, osteoarthritis, musculoskeletal pain

## Abstract

Background

The purpose of this study was to investigate the associations among knee osteoarthritis severity, body mass index, and physical functions in Saudi Arabian adults.

Methodology

In this multi-center, cross-sectional study, we performed a secondary data analysis that included 189 adults aged 55 years or above with doctor-diagnosed knee osteoarthritis enrolled in five hospitals in Riyadh, Saudi Arabia, between March 2016 and March 2017. According to knee osteoarthritis severity, all of the individuals were divided into the following three groups: mild (n = 36), moderate (n = 75), and severe (n = 78). A high body mass index was defined as a body mass index score of >25 kg/m^2^. Physical function was evaluated using the 36-item physical functioning subscale.

Results

Severe knee osteoarthritis had a significantly 6.47-fold (95% confidence interval (CI) = 2.95-14.22, p < 0.0001) higher risk of physical function than those with mild knee osteoarthritis after adjusting for age, sex, educational status, occupational status, affected knee with osteoarthritis, knee pain, and body mass index. However, moderate knee osteoarthritis had a 1.22-fold higher risk of physical function, but the association was not statistically significant (95% CI = 0.60-2.49, p = 0.578).

Conclusions

Severe but not moderate knee osteoarthritis was more likely to have the worst physical function than mild knee osteoarthritis among adults with a high body mass index in Saudi Arabia.

## Introduction

Knee osteoarthritis (KOA) is a significant condition with pain as the primary symptom that causes difficulty in physical functioning and health-related quality of life (QoL) in the aging population [1]. The most common practical problem in KOA is pain-induced physical function and mobility limitation, especially walking ability and stair negotiation [2]. In such circumstances, developing effective pain management treatment options is essential for those with KOA, especially those who have a higher body mass index (BMI) [3]. A recent study found that older persons with KOA performed considerably longer in completing the Sit-to-Stand and Timed Up-and-Go tests and had significantly shorter one-leg standing times and lower maximal walking speeds [4]. According to the study, they also had a much inferior QoL and poor physical and social roles.

KOA and a higher BMI are rapidly growing health problems and significant causes of adverse health outcomes in many nations, including Saudi Arabia, particularly in people aged over 50 [5]. The KOA problem in Saudi Arabia differs from Western culture in their need for full knee flexion for daily activities, such as praying, ablution, and sitting to eat on the floor [6]. A recent study, however, revealed that function and QoL among Saudi Arabian Muslims have worsened during the nine years of modified (chair-use) authentic Islamic prayer [6]. Furthermore, a higher BMI has become more prevalent in the Middle East over the past 20 years, particularly in Saudi Arabia, which ranked third among Middle Eastern nations after Kuwait and Iraq [7]. One explanation could be that those over 40 of age had the highest prevalence of obesity, which grows until age 70 and significantly corresponds with aging [8].

In a recent study [5], an associated modifiable risk factor, such as a high BMI, has been linked to an increased risk of KOA in Saudi Arabia. Another recent study found that obesity is linked to a higher risk of multisite pain in the lower limbs [9]. Furthermore, much literature has shown the relationship between a high BMI and physical function [10]. In contrast, multiple studies have also shown that adults with a higher BMI report more physical function impairment than healthy ones [11]. It seems possible that having a higher BMI may increase the burden on a knee, which significantly increases the severity of knee pain [12]. However, the relationship between severe radiographic KOA and physical function has been examined limitedly among adults with a high BMI in the Saudi Arabian context [13]. Such additional research is required in this context because it has been widely noted that age-related severe KOA is one of the reasons why older Muslims around the world now perform prayers on chairs rather than the ground as is customary in mosques and at home [6]. Furthermore, this severe KOA has caused these older Muslims who are used to praying in chairs to experience a decline in their QoL [6]. Additionally, a higher BMI has become more common over the past 20 years, particularly in Saudi Arabia [7].

Therefore, the purpose of this study was to investigate the association among KOA severity, BMI, and physical functions in Saudi Arabian Adults. According to the study’s hypothesis, persons with severe KOA, but not moderate KOA, were more likely than adults with mild KOA to have the lowest physical function among adults with a high BMI. The findings may point to the need for Saudi Arabian authorities to monitor population-wide KOA and BMI metrics in addition to the aforementioned treatment suggestions for patients with severe KOA to meet the goals of the nation’s 2030 vision, which include a vibrant society and a better standard of living [14].

## Materials and methods

Study design and setting

In this cross-sectional study, we performed a secondary data analysis using data that was primarily collected for another purpose from individuals who visited the orthopedic and physiotherapy departments of five clinics for treatment in Riyadh, Saudi Arabia between March 2016 and March 2017 [12]. The clinics were King Saud University Medical City (KSUMC), King Faisal Specialist Hospital & Research Center (KFSHRC), King Saud Medical City (KSMC), Dwadmi General Hospital (DGH), and Quwaieah General Hospital (QGH). The original study was conducted as per the Declaration of Helsinki rules and approved by the Institutional Review Boards (IRB) of the KSUIMC (CAMS 143-36/37), KFSHRC (ORA/1171/37), KSMC (H-01-R-053), DGH (H-01-R-012), and QGH (H-01-R-012). Informed consent was obtained from all individuals involved in the study.

IRB approval was waived for the current study because a secondary analysis was done using the initial data that were gathered for a different objective. The lack of any identifying information in the data and the fact that the codes cannot be accessed owing to good coding can be used to explain this. There was no need for informed consent for the ongoing investigation. A secondary study of previously gathered data cannot be used to pinpoint a specific individual.

Study participants

A total of 189 males and females aged 55 years and above with a doctor-diagnosed radiographic KOA according to the American College of Rheumatology standards [15] were recruited from orthopedic or physiotherapy departments of the above-stated five hospitals. We excluded patients with a healthy weight, severe rheumatoid arthritis or fractures, and those who had received significant surgery on lower limbs or an intra-articular injection in the last six months (n = 7). We also excluded missing data (n = 14) from the analysis.

Physical function

Physical function at each site was assessed using the 10-item Physical Functioning (10-PF) subscale of the Arabic 36-item Short Form Health Survey (SF-36) [16]. The PF-10 consists of 10 items that evaluate the extent of health-related limitations in a variety of physical activities, such as vigorous, moderate, lifting or carrying groceries, climbing several flights of stairs, climbing one flight of stairs, bending, kneeling, or stooping, walking more than one minute, walking several blocks, and both or dressing. The scoring of the PF-10 was based on Likert’s method for summated rating scales in which the algebraic sum of the 10 item scores (1 = limited a lot; 2 = limited a little; 3 = not limited at all) was computed. Raw scores were summed and linearly transformed into a 0-100 scale, with 100 indicating the most favorable level of physical functions. The reliability and validity of the PF-10 across patient groups have been well-established elsewhere [16].

Knee osteoarthritis severity

We used an exposure of KOA severity defined according to the Kellgren-Lawrence (KL) scale proposed by Kellgren and colleagues [17]. The radiograph grade (0-4) corresponded to the severity of KOA, with grade 1 denoting mild OA, 2-3 denoting moderate OA, and grade 4 denoting severe OA. All patients were divided into the following three groups based on their KOA severity (KL): mild (n = 36), moderate (n = 75), and severe (n = 78). The KL scale for KOA has already been proven valid and reliable, with a mean area under the curve of 0.92 signifying excellent [17]. The BMI was calculated by dividing weight in kilograms (kg) by square meters (m^2^) height. A high BMI was defined as a BMI score greater than 25 kg/m^2^ [18].

Covariates

We collected sociodemographic and clinical variables, such as age, gender, education, occupation, affected knee with OA, BMI, duration of KOA in a year, and visual analog scale. The following variables were dichotomized: gender (males and females), education (primary school or less and high school or more), occupation (employed and self-employed or retired), and affected knee with OA (right/left and both). Other covariates were used as continuous because of the small number of samples in each category.

Statistical analysis

The Farrington-Manning test was used to calculate the needed sample size per group to establish valid results using the level of significance (alpha = 0.05), power (0.80), and proportion between groups (0.23, 0.11). The necessary minimum sample per group was found to be 32. A Shapiro-Wilk test was utilized to determine data normality. The descriptive means and standard deviation (SD) statistics were calculated for continuous variables. At the same time, count and percentage were computed for categorical variables for patients in the following three groups: mild, moderate, and severe radiographic KOA. The analysis of variance was used to show significant differences between the groups. Logistic regression analysis was applied to assess physical function in adults with severe and moderate KOA than those with mild KOA who were overweight or obese.

The association was examined with unadjusted and adjusted analyses. In unadjusted analysis, the association was examined with no other covariates. In adjusted analysis, the association was tested with covariates, such as age, sex, education status, employment status, affected knee with KOA, and knee pain. Mild KOA was used as a reference. The values from the analyses were shown as odds ratios (ORs) and 95% confidence intervals (95% CIs). All analyses were performed using the statistical analysis software (SAS) version 9.2 (SAS Institute, Inc., Cary, NC, USA) for Windows.

## Results

**Figure 1:**
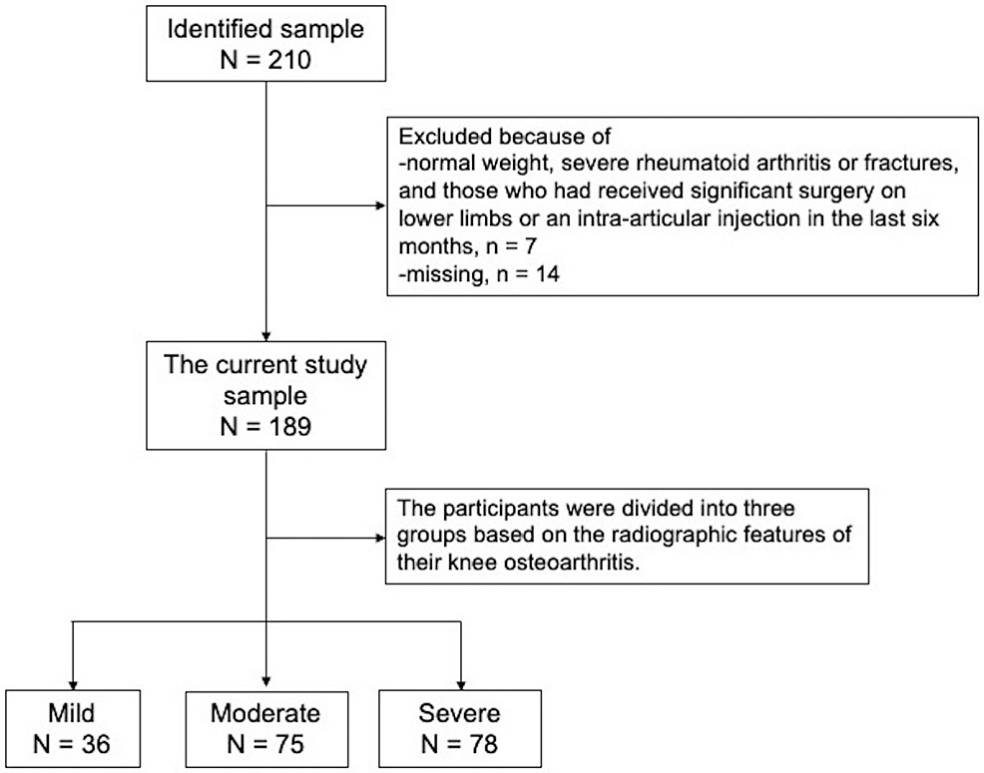
Flowchart of the study sample. Note: The Kellgren-Lawrence (KL) scale with grade 1 denoting mild knee osteoarthritis, grades 2-3 denoting moderate knee osteoarthritis, and grade 4 denoting severe knee osteoarthritis.

The flowchart of the study sample is illustrated in Figure 1. Of the 189 patients with KOA, 36 had mild concerns (19%), 75 had moderate issues (40%), and 78 had severe concerns (41%) based on their radiographic features of KOA.

Compared to individuals with mild and moderate KOA, those with extreme KOA were three years older. In patients with advanced KOA, females (78%) predominated as the dominant sex. Patients with severe KOA were more likely to be self-employed or retired (77%) and had less education (53%) than other patients. For patients with severe KOA, the average time since the onset of the condition and the amount of pain was more than eight years and 7 points, respectively (Table 1).

**Table 1:** Descriptive characteristics of the total sample (n = 189). Values are presented as the mean ± standard deviation or count (percentage). OA = osteoarthritis; VAS = visual analog scale

Characteristics	Radiographic features of knee osteoarthritis			P-value
	Mild, N = 36 (19%)	Moderate, N = 75 (40%)	Severe, N = 78 (41%)	
Age in years	56.3 ± 7.4	56.9 ± 8.0	59.8 ± 9.2	0.049
Sex				0.0006
Male	21 (58)	27 (36)	17 (22)	
Female	15 (42)	48 (64)	61 (78)	
Education				<0.0001>
Primary school or less	7 (19)	17 (23)	41 (53)	
High school or more	29 (81)	58 (31)	37 (47)	
Occupation				0.345
Employed	13 (36)	20 (27)	18 (23)	
Self-employed or retired	23 (64)	55 (73)	60 (77)	
Affected knee with OA				0.091
Right/left	11 (31)	11 (15)	12 (15)	
Both	25 (69)	64 (85)	66 (85)	
Body mass index (kg/m^2^)	33.6 ± 6.8	32.5 ± 4.1	33.8 ± 5.2	0.290
Duration of knee OA in years	2.2 ± 2.2	4.0 ± 3.3	8.3 ± 5.3	<0.0001>
VAS for pain	4.1 ± 1.7	5.3 ± 1.6	7.6 ± 1.6	<0.0001>
Mobility-related disability	51.6 ± 19.2	46.4 ± 15.0	32.8 ± 9.6	<0.0001>

**Figure 2:**
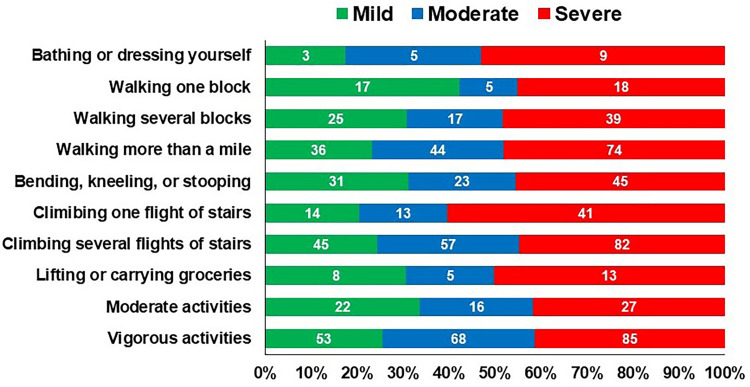
The proportion of impaired physical function distribution according to the mild, moderate, and severe knee osteoarthritis. Note: The numbers under the bars represent the percentage.

Figure 2 shows the distributions of physical function for mild, moderate, and severe KOA. Individuals with severe KOA had a higher percentage of difficulty in physical function compared to those with mild and moderate KOA.

Severe KOA had a significantly 9.88-fold (95% CI = 4.68-20.85, p < 0.0001) higher risk of physical function among adults with a high BMI than those with mild KOA. After adjusting for all variables, the association remained substantial and statistically significant (AOR = 6.47, 95% CI = 2.95-14.22, p < 0.0001). Moderate KOA had a 1.54-fold (95% CI = 0.77-3.09) risk of physical function among adults with a higher BMI than those with mild KOA, but was not statistically significant (p = 0.218). The association remained not statistically significant after adjusting for all covariates (OR = 1.22, 95% CI = 0.60-2.49, p = 0.578) (Table 2).

**Table 2:** Regression analysis of the association between the radiographic features of knee osteoarthritis and physical functions (n = 189). Unadjusted, groups without covariates. Adjusted, unadjusted with adjusted for age, sex, educational status, occupational status, affected knee with knee osteoarthritis, knee pain, and body mass index. OA = osteoarthritis; OR = odds ratio; AOR = adjusted odds ratio; CI = confidence interval

Radiographic feature of knee osteoarthritis	Unadjusted			Adjusted		
	OR	95% CI	P-value	AOR	95% CI	P-value
Mild	1.00 (reference)			1.00 (reference)		
Moderate	1.54	0.77–3.09	0.218	1.22	0.60–2.49	0.578
Severe	9.88	4.68–20.85	<0.0001>	6.47	2.95–14.22	<0.0001>

## Discussion

This study investigated the physical function of Saudi Arabian adults with severe and moderate KOA compared to those with mild KOA who all had a high BMI. The findings show that among Saudi Arabian adults with high BMI, severe, but not moderate, KOA was more likely to be linked to a higher risk for physical function than mild KOA, even after adjusting for sociodemographic and clinical characteristics. The insufficient physical activity brought on by severe KOA, regardless of a high BMI, may contribute to a higher risk for physical function in this population. A recent national survey [19] found that most Saudi adults selected from 26,000 families of 13 administrative regions across Saudi Arabia had low physical activity levels.

Most participants in this study, particularly females, had moderate-to-severe KOA. KOA had an average duration of 4.8 years and a pain score of 5.7 on the VAS scale. Adults with severe KOA also exhibited a serious mobility-related impairment. These results are pertinent to the most recent cross-sectional population research conducted in Saudi Arabia [5]. This study found that the total prevalence of KOA was 18.9%, and females were substantially more affected than males. The study reported that age, sex, prior injuries, and obesity may be linked to KOA in the Saudi Arabian population.

The results of this study are consistent with our earlier findings, which showed that even after adjusting for sociodemographic variables, severe KOA was still strongly linked to more significant pain and decreased health-related QoL [12]. Obesity was strongly related to slow gait speed in people with recurrent knee pain and reduced over time [11]. This result mostly corroborates the findings of past research that showed that the interaction of obesity, rheumatoid arthritis, and other rheumatic illnesses greatly impacted various health-related QoL measures and objectively measured physical function [20]. A recent cross-sectional study [6] evaluated the QoL and knee difficulty associated with KOA in older Muslims recruited from 27 mosques who had converted to chair usage, with an average of five individuals offering prayers in place of the traditional Muslim prayer positions of kneeling (both thigh and calf contact) and squatting. The findings showed that function and QoL had decreased over the past nine years of modified (chair-use) prayer among Saudi Arabian Muslims.

This study’s results align with previous studies, demonstrating that physical function in older Japanese individuals varied according to overweight or obesity [10] and that males with a high BMI had considerably shorter one-leg standing periods with open eyes. According to another prospective cohort study [21], obese senior males had the highest risk of physical function decline. The sex diversity in physical function risk was not sufficiently demonstrated in this study. The fact that numerous musculoskeletal issues have a negative association with higher BMI [22] may be a plausible physiological or biomechanical understanding supporting the findings of the current investigation. With mechanical strain and metabolic inflammation, KOA patients with a higher BMI eventually develop severe KOA [22]. This was due to accelerated cartilage destruction. As a result, physical function was affected. According to a study, cortisol production is positively correlated with pain intensity connected to KOA [23], and less cortisol is produced in response to less pain.

This study’s findings may assist clinicians, researchers, and policymakers in comprehending the connection between severe KOA and physical function in the obese or overweight adult population in Saudi Arabia. These people may benefit from moderate exercise thrice weekly to reduce pain and improve function [24]. According to a recent study [25], physical exercise, especially at high levels, had a favorable association with less severe KOA. However, the participants in this cross-sectional study had a mean age of 44.3 years, and 84.1% were female. Most had severe KOA that persisted for at least five years. According to another study [26], independent of age, BMI, and physical activity, the study indicated that among males and females with severe KOA who received unilateral TKA, greater serum testosterone levels were related to decreased pain in the operated knee. In addition, the study discovered that regardless of age, BMI, or physical activity, greater serum testosterone levels were linked to decreased disability in females knees who had undergone surgery and those who had not.

The current study’s strength was that it was multicenter and used the Arabic version of the 10-PF subscale of SF-36, a commonly used and validated tool to evaluate physical function. The American College of Rheumatology’s diagnostic standards were also used to diagnose KOA. These findings might be slightly constrained by a cross-sectional design. Therefore, it is important to interpret these results carefully. This study’s self-report of physical function has another drawback. It is essential to be aware of any potential bias in those self-reports. Lastly, the sample could not represent Saudi Arabia’s entire KOA patient population. However, our findings might still apply to patients from the Riyadh region.

## Conclusions

This study aimed to investigate the relationship among KOA severity, BMI, and physical function in Saudi Arabian adults with a higher BMI. The results indicate severe but not moderate KOA more likely had the worst physical function than mild KOA among adults with a high BMI. The results presented here provide new insights into creating rehabilitation strategies, such as managing musculoskeletal pain through weight loss exercise programs. More extensive national research is needed to learn about the physical function limitations in this patient population.
